# Rescue In Vitro Maturation of a Germinal Vesicle Oocyte Leading to Pregnancy in a Case of Unexplained Infertility

**DOI:** 10.7759/cureus.92989

**Published:** 2025-09-23

**Authors:** Elham A Akbari, Zakwan Khrait, Natalia Kondakova, Steven Eaton

**Affiliations:** 1 Reproductive Medicine and Infertility, Dubai Health Authority, Dubai, ARE; 2 Obstetrics and Gynecology, IVF International Fertility Center, Dubai, ARE; 3 Reproductive Medicine and Infertility, IVF International Fertility Center, Dubai, ARE; 4 Embryology, IVF International Fertility Center, Dubai, ARE

**Keywords:** case report, controlled ovarian stimulation, euploid embryo, gv oocytes, icsi, ivf, pgt-a, pregnancy, rescue ivm

## Abstract

Rescue in vitro maturation (rescue-IVM) refers to the post-retrieval culture of immature oocytes - typically at the germinal vesicle (GV) or metaphase I (MI) stage - under laboratory conditions, allowing them to complete maturation to metaphase II (MII) and become suitable for fertilization.

This case report presents a successful pregnancy following rescue-IVM and preimplantation genetic testing for aneuploidy (PGT-A) in a 35-year-old woman with unexplained infertility and a history of ectopic pregnancy.

Immature GV-stage oocytes retrieved during in vitro fertilization (IVF) were cultured using standard protocols, and several progressed to MII without pharmacologic stimulation. Following delayed intracytoplasmic sperm injection (ICSI), two euploid blastocysts were obtained, one of which led to a viable singleton pregnancy.

This case underscores the clinical relevance of rescue-IVM in IVF cycles with high proportions of immature oocytes, particularly in patients with diminished ovarian reserve or tubal factor infertility. It highlights the importance of individualized assisted reproductive technology (ART) strategies, and the potential of advanced embryology techniques - such as time-lapse monitoring and optimized ICSI timing - to improve outcomes in poor-prognosis cases.

## Introduction

Rescue in vitro maturation (rescue-IVM) of germinal vesicle (GV) oocytes retrieved during stimulated in vitro fertilization (IVF) cycles has gained increasing attention as a potential contributor to embryo yield, particularly in patients with a high proportion of immature oocytes. Unlike conventional IVM, which involves dedicated culture protocols and pharmacologic stimulation, rescue-IVM refers to the post-retrieval culture of immature oocytes under standard laboratory conditions, allowing them to complete maturation without hormonal intervention. Although these oocytes were historically considered suboptimal, emerging evidence suggests that, under appropriate conditions, they may achieve both nuclear and cytoplasmic maturity sufficient for fertilization and blastocyst development [1,2].

Morphological characteristics such as cytoplasmic granulation have been proposed as predictors of maturation potential and developmental competence in rescue-IVM settings [3]. Rescue maturation strategies, where GV oocytes are cultured after retrieval, have demonstrated variable success depending on media composition, incubation timing, and laboratory protocols [4]. While maturation rates and fertilization outcomes may be lower than those of in vivo matured oocytes, the inclusion of rescue-IVM oocytes in IVF protocols may offer a meaningful increase in embryo availability, particularly in low-yield cycles or poor-prognosis patients.

In parallel, preimplantation genetic testing for aneuploidy (PGT-A) has become a widely adopted tool for embryo selection. PGT-A has been shown to reduce miscarriage rates and improve implantation success, especially in women over 35 years of age [5,6]. However, its benefit in younger patients and its impact on cumulative live birth rates remain subjects of ongoing debate [7]. Recent studies suggest that, when used judiciously, PGT-A may shorten time to pregnancy and improve clinical outcomes, particularly when integrated into individualized assisted reproductive technology (ART) strategies [8].

Together, these developments underscore the importance of re-evaluating the role of immature oocytes and advanced genetic screening in modern IVF practice. Continued refinement of laboratory techniques and patient-specific protocols may expand the utility of rescue-IVM and optimize reproductive success.

## Case presentation

A 35-year-old nulliparous woman presented to our fertility center with a two-year history of unexplained infertility. Her medical history included a laparoscopic left salpingectomy following an ectopic pregnancy. She underwent controlled ovarian stimulation for IVF with intracytoplasmic sperm injection (ICSI) and PGT-A. The stimulation protocol included recombinant follicle-stimulating hormone (rFSH, 300 IU daily), initiated on cycle day 3 for nine days, with a gonadotropin-releasing hormone (GnRH) antagonist introduced on day 7. Final oocyte maturation was triggered using a combination of choriogonadotropin alfa and GnRH agonist 36 hours prior to retrieval.

**Figure 1:**
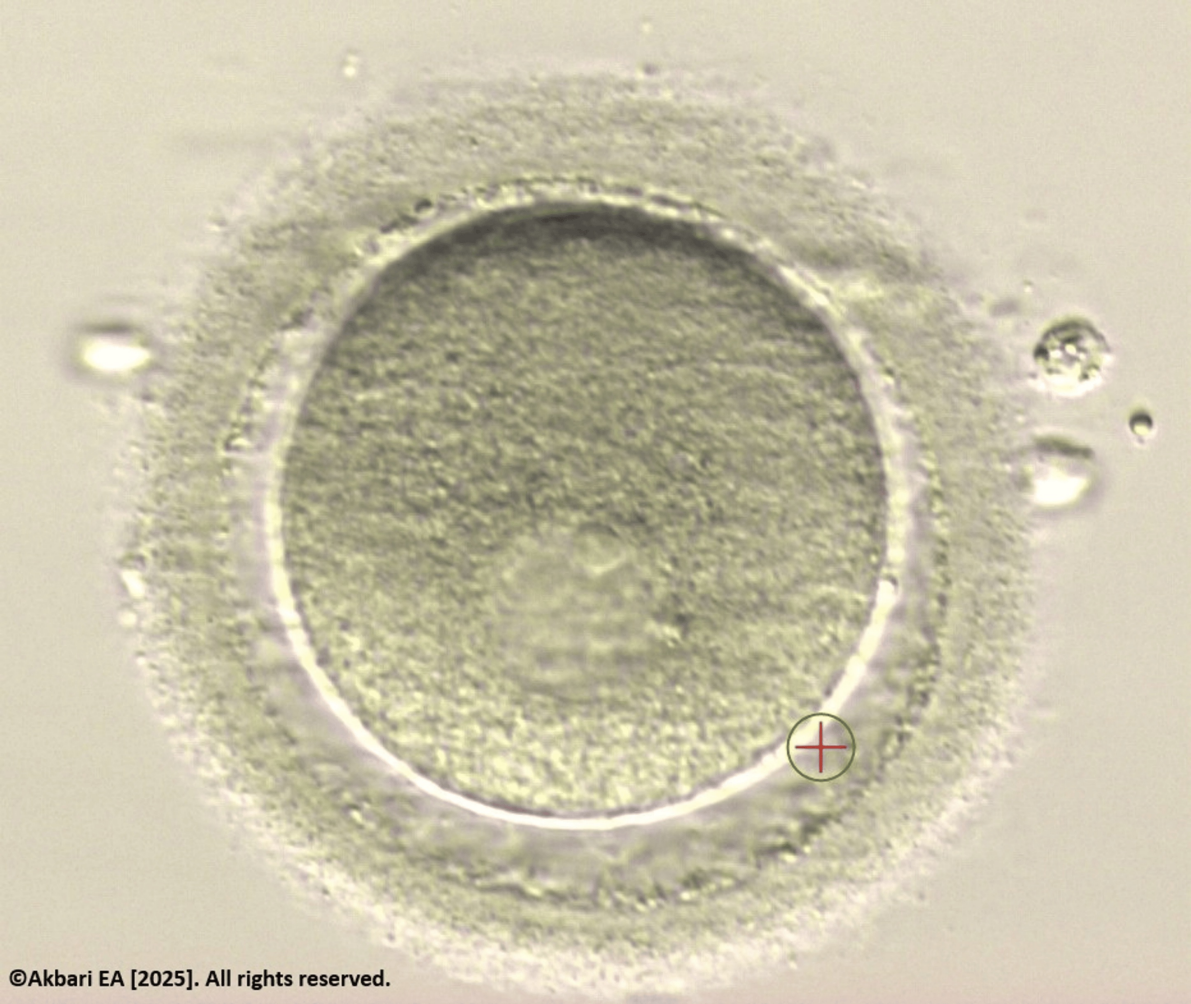
Germinal vesicle (GV) stage oocyte Original image created by the author, ©Akbari EA 2025.

Transvaginal oocyte retrieval on cycle day 13 yielded 12 oocytes: six mature (metaphase II, or MII) and six immature at the GV stage (Figure 1). The immature oocytes were cultured using a rescue-IVM protocol under standard laboratory conditions without pharmacologic supplementation. All GV oocytes were placed in a time-lapse incubator to monitor nuclear maturation dynamics and polar body extrusion. Within 24 hours, four oocytes reached MII, confirmed by the presence of the first polar body (PB1).

**Figure 2:**
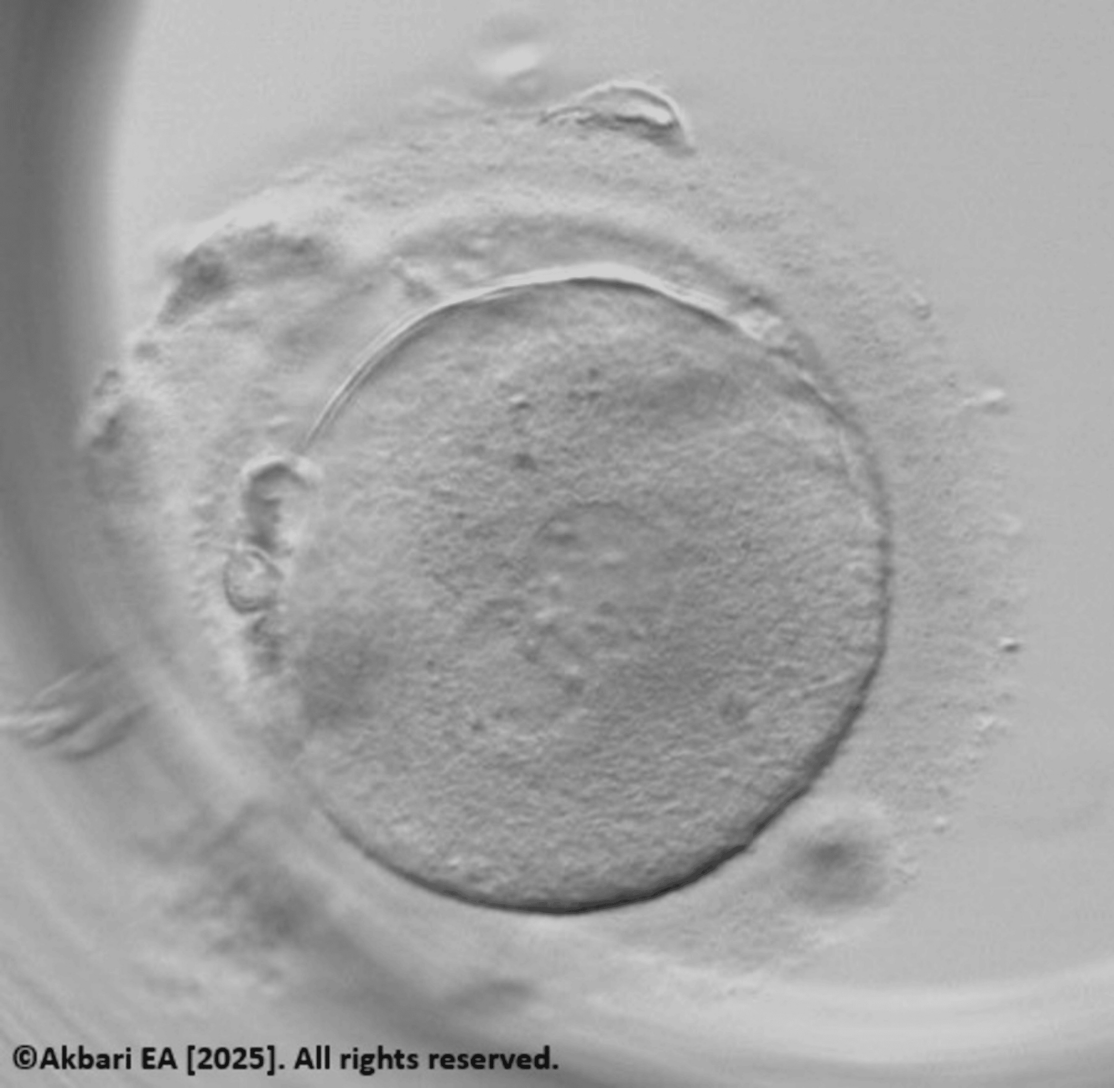
Rescue in vitro maturation (IVM) derived embryo at zygote stage Original image created by the author, ©Akbari EA 2025.

**Figure 3:**
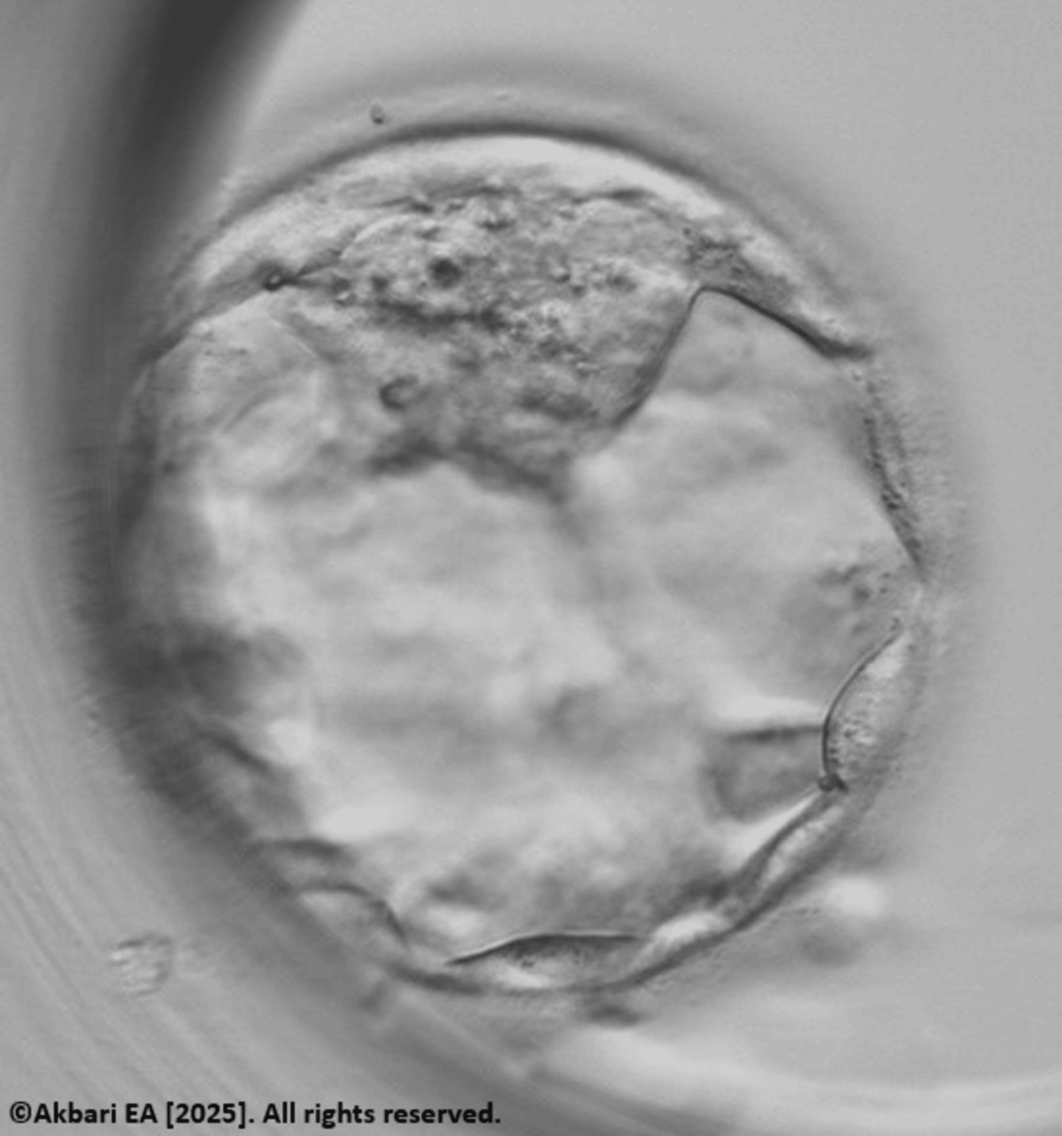
Rescue in vitro maturation (IVM) derived blastocyst before biopsy for preimplantation genetic testing for aneuploidy (PGT-A) Original image created by the author, ©Akbari EA 2025.

To optimize fertilization potential, ICSI was performed approximately six to eight hours after polar body extrusion, allowing sufficient time for cytoplasmic maturation. The resulting zygotes were monitored for pronuclear formation and cleavage (Figure 2). Of the four rescue-IVM oocytes injected, two developed into blastocysts graded 5BB and 4AB. Both embryos were confirmed euploid via PGT-A, demonstrating developmental competence comparable to their in vivo matured counterparts (Figure 3).

**Figure 4:**
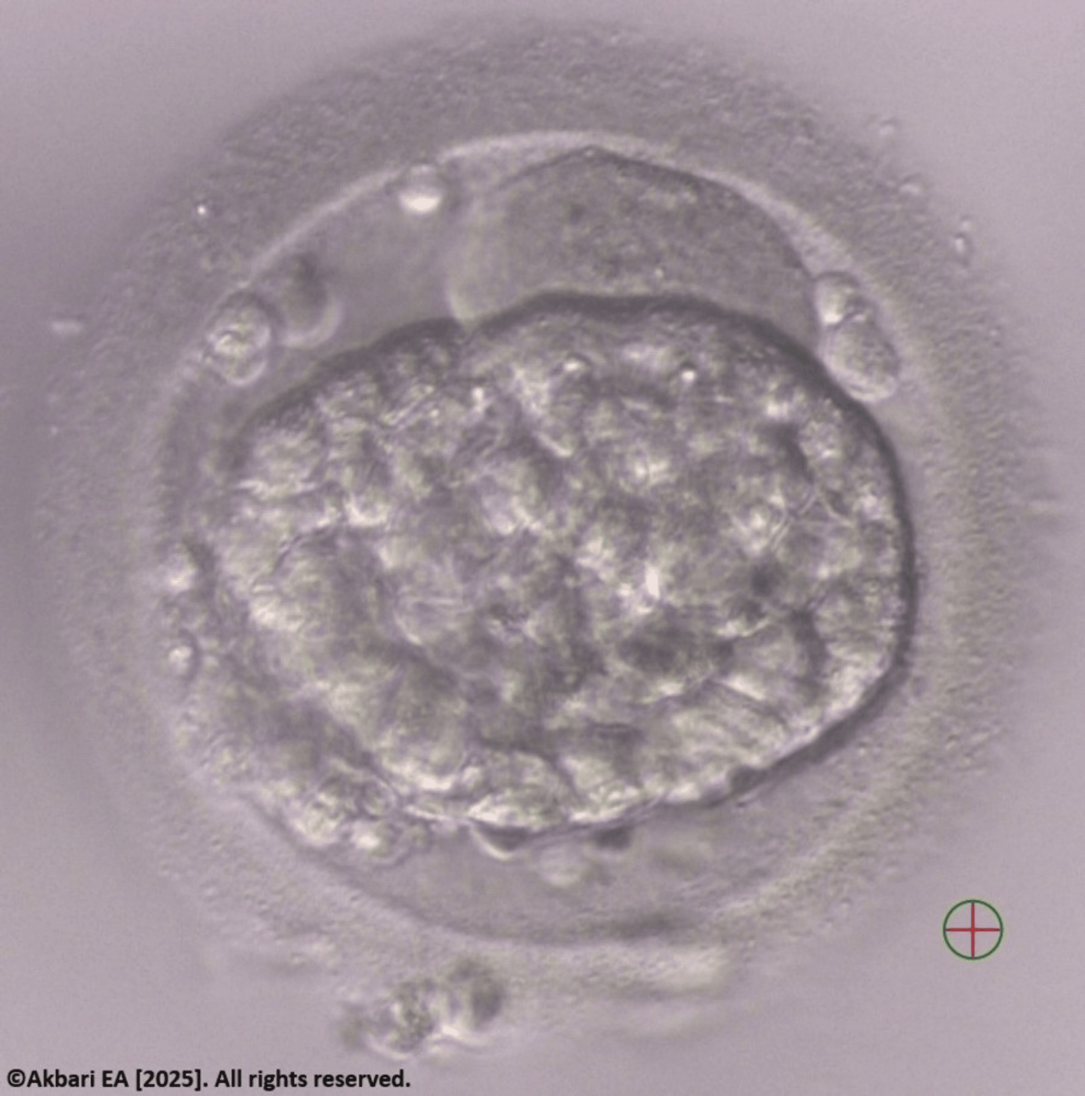
Rescue in vitro maturation (IVM) derived embryo immediately after thawing Embryo transfer was performed two hours after full re-expansion. Original image created by the author, ©Akbari EA 2025.

**Figure 5:**
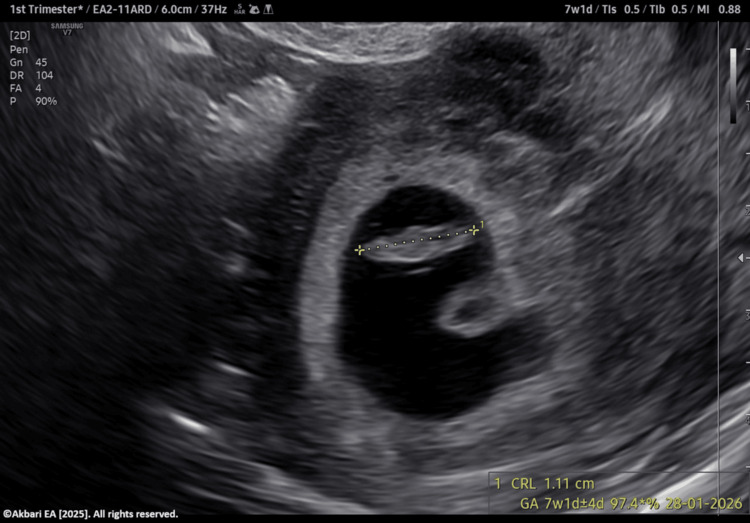
Transvaginal ultrasound image showing crown rump length (CRL) of seven weeks and one day Original image created by the author, ©Akbari EA 2025.

**Figure 6:**
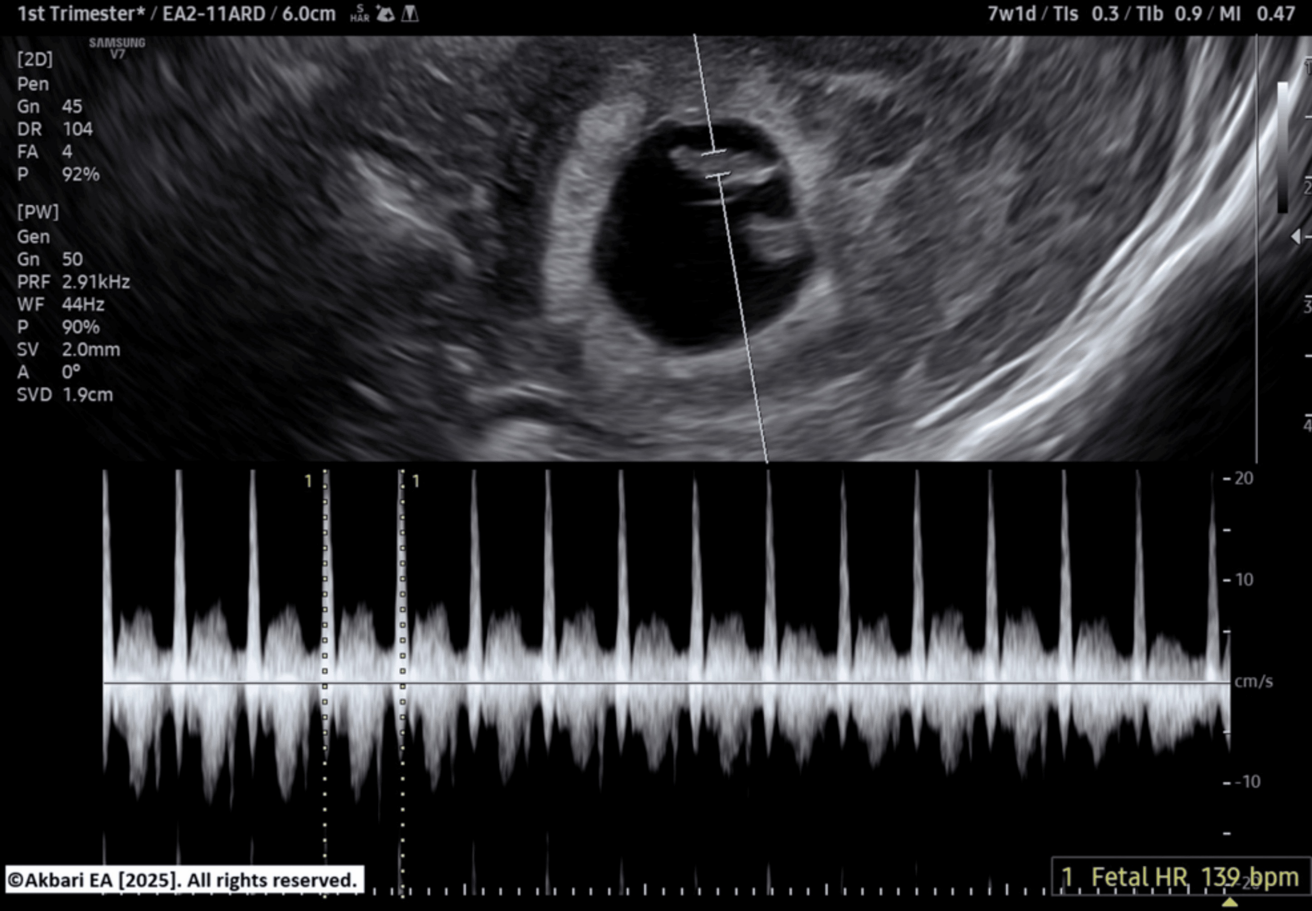
Transvaginal ultrasound image showing positive fetal heart at seven weeks of gestation Original image created by the author, ©Akbari EA 2025.

The patient elected to cryopreserve all embryos and defer transfer. Endometrial preparation for frozen embryo transfer was initiated at a later date. A single euploid blastocyst was thawed and evaluated prior to transfer (Figure 4). Progesterone supplementation was provided via oral, vaginal, and intramuscular routes. Serum β-hCG measured on day 8 post-transfer was positive. A transvaginal ultrasound at seven weeks’ gestation confirmed a viable singleton pregnancy (Figures 5-6). The patient was subsequently discharged to routine obstetric care.

## Discussion

This case highlights the successful application of rescue-IVM combined with PGT-A in a patient with unexplained infertility and prior tubal surgery. The ability to recover and mature oocytes that would otherwise be discarded during conventional IVF represents a meaningful advancement in ART, particularly for patients with limited reproductive options.

Rescue-IVM refers to the practice of culturing immature oocytes - typically at the GV or metaphase I (MI) stage - for 24 to 48 hours post-retrieval, allowing them to complete maturation to MII. Once matured, these oocytes can be fertilized via ICSI and cultured to the blastocyst stage [9-11]. In this case, six GV oocytes were cultured under standard laboratory conditions, four of which reached MII and contributed to the formation of two euploid blastocysts, ultimately resulting in a viable pregnancy.

Rescue-IVM is particularly relevant in several clinical scenarios: poor responders with few mature oocytes retrieved, cycles with unexpectedly high proportions of immature oocytes, and cases where maximizing embryo yield is critical - such as before discontinuing treatment. Although oocytes matured via rescue-IVM often exhibit lower fertilization and developmental competence compared to those retrieved at MII, emerging evidence suggests that euploid embryos derived from rescue-IVM can implant and lead to healthy live births [2,12].

A key consideration in rescue-IVM is the timing of ICSI following the extrusion of the PB1, which marks nuclear maturation. However, cytoplasmic maturation - essential for spindle stabilization and organelle redistribution - often lags behind. Fertilizing too early may compromise embryo development. Studies recommend delaying ICSI by four to six hours post-PB1, with some protocols extending to 6 to 10 hours, especially for GV-origin MII oocytes [13,14]. Time-lapse imaging systems enhance precision by allowing embryologists to monitor PB1 extrusion and optimize insemination timing.

While rescue-IVM remains an experimental approach, its clinical utility is gaining recognition. Professional societies emphasize the importance of individualized protocols and informed consent, given the variability in outcomes across centers. Optimized culture systems, including co-culture with ovarian support cells, are under investigation to improve maturation quality [15,16]. Additionally, refining laboratory protocols - such as delayed ICSI and extended culture to the blastocyst stage - may elevate the chances of obtaining euploid embryos and achieving clinical pregnancy.

The integration of PGT-A in this case further strengthened the strategy by enabling the selection of chromosomally normal embryos. Although its benefit in younger patients remains debated, PGT-A has been shown to reduce miscarriage rates and improve implantation success in select populations, particularly those with complex reproductive histories or limited embryo yield [17,18].

## Conclusions

This case underscores the clinical utility of rescue-IVM as a viable strategy in IVF cycles with a high proportion of immature oocytes. By salvaging GV oocytes and enabling their maturation and fertilization, rescue-IVM enhances oocyte utilization and contributes to the generation of euploid embryos with implantation potential comparable to those matured in vivo - particularly when paired with precise ICSI timing and PGT-A.

Time-lapse incubation provided valuable insights into maturation kinetics, informing optimal ICSI timing and improving embryo selection. These findings support the integration of rescue-IVM into protocols for patients with asynchronous follicular development or diminished ovarian reserve, where maximizing embryo yield is critical. Standardization of laboratory conditions and maturation monitoring will be essential to ensure reproducibility and optimize clinical outcomes. A key limitation of this article is its lack of generalizability, as it is based on data from a single patient.
